# Effect of auto flash margin on superficial dose in breast conserving radiotherapy for breast cancer

**DOI:** 10.1002/acm2.13287

**Published:** 2021-05-24

**Authors:** Lu Wang, Gang Qiu, Jianhe Yu, Qunhui Zhang, Li Man, Li Chen, Xiaoxiao Zhang, Qun Ren, Hongxia Xu, Xiaolong Hua

**Affiliations:** ^1^ Department of Radiotherapy Anshan Cancer Hospital Anshan Liaoning China; ^2^ Department of Oncology Ward 2 Hebei General Hospital Shijiazhuang Hebei China; ^3^ Department of Oncology Ward 3 Xinghua People's Hospital Taizhou Jiangsu China; ^4^ Surgical oncology Anshan Cancer Hospital Anshan Liaoning China; ^5^ Medical oncology Anshan Cancer Hospital Anshan Liaoning China; ^6^ Department of Radiotherapy Xinghua People's Hospital Taizhou Jiangsu China

**Keywords:** auto flash margin, breast cancer, inspiratory exercise, superficial dose, XVMC

## Abstract

**Purpose:**

To investigate the dose‐effect of Auto Flash Margin (AFM) on breast cancer's superficial tissues based on the Treatment Planning System (TPS) in the breast‐conserving radiotherapy plan.

**Methods:**

A total of 16 breast‐conserving patients with early stage breast cancer were selected, using the X‐ray Voxel Monte Carlo (XVMC) algorithm. Then, every included case plan was designed using a 2 cm‐AFM (the value of AFM is 2 cm) and N‐AFM (without AFM). Under the condition of ensuring the same configuration of #MU and collimator, the absorbed dose after a simulated inspiratory motion was calculated again using the new plan center, which moved backward to the linac source. The dose difference between the measurement points between AFM and N‐AFM groups was compared.

**Results:**

In the dose results, PTV_V50Gy_ of the AFM group was superior to that of the N‐AFM group, PTV_D2%_, PTV_Dmean_, Lung_Ipsi_V20Gy_, Lung_Ipsi_Dmean_, and Body_Dmax_. Also, the dose results of the N‐AFM group were significantly higher than those of the AFM group. However, there was no significant difference between Lung_Contra_V5Gy_, Heart_Dmean_, and Breast_Contra_V10Gy_ in the two groups. In the collimator alignments at the same angle between groups, the AFM group formed an apparent air region outside the collimator compared with the N‐AFM group. In the XVMC algorithm feature parameter, the AFM group had less #MU, higher QE, and slightly longer optimization time. The #segments of both groups were close to the 240 control points preset by the plan. The validation results of EBT3 film in both groups were more significant than 95%, meeting the clinical plan's application requirements. The difference in film results between groups was mainly reflected in the dose distribution at the near‐source. 4DCT was used to summarize the maximum and minimum inspiratory motion distances of 7.31 ± 0.45 and 3.42 ± 0.91 mm respectively.

**Conclusions:**

These results suggest that the AFM function application could significantly reduce the possibility of insufficient tumor target caused by inspiratory motion and ensure sufficient tumor target exposure.

## 1 INTRODUCTION

Breast cancer is one of the most common malignant tumors in women in both developed and developing countries. However, the survival time of these patients is generally longer. Moreover, under the influence of psychological factors, these patients are inclined to accept treatment that is a combination of minimally invasive surgery and postoperative breast‐conserving radiotherapy. Breast cancer is a superficial tumor that is difficult to remove by surgery completely, so radiotherapy is necessary. To perform radiotherapy in breast cancer patients who have undergone breast‐conserving surgery, adequate exposure of the superficial area can effectively reduce the probability of recurrence of the tumor *in situ*.^1^, ^2^, ^3^ With recent developments in radiotherapy technology, breast‐conserving surgery, and intensity‐modulated radiotherapy (IMRT) has been widely used for the treatment of breast cancer due to its apparent advantages such as dose conformation outside the tumor target, dose uniformity within the tumor target, and dose control of the organs at risk (OARs).

Currently, the management and control technique of inspiratory motion in the superficial area of breast cancer is mostly to make up for the off‐target irradiation of the chest wall by expanding PTV or adding a virtual bolus. However, for high‐precision radiotherapy techniques like 3DCRT and IMRT, the tumor target location's deviation is likely to result in a higher dose, making the surrounding organs at risk. Besides, the virtual bolus will misjudge the actual absorbed amount in the superficial breast area and affect the radiotherapy dose's accuracy.^4^, ^5^ AFM function is a tool that produces reasonable monitor units per fraction and a small standard deviation in a plan. Application of this tool provides a uniform distribution in the buildup region. In the current common IMRT schemes for breast cancer, the radiation field is formed according to the shape of the target area. In this case, off‐target irradiation due to inhalation motion cannot be avoided. After applying the AFM function, a cavity within the radiation exposure range is established outside the body surface, so even under the inhalation motion, the superficial tissues can still be guaranteed to be irradiated.

## 2 MATERIALS AND METHODS

### 2.A Data

In this study, we enrolled 16 patients (nine patients with T1N0M0 and seven patients with T_2_N_0_M_0_, aged 23‐45 years) whose left breast had cancer and underwent breast‐conserving surgery. Here, we first used a 4D computerized tomography (CT) scan ( ie "beam ‐ step ‐ beam," a technique for performing continuous multiphase CT scans, CT rotation time was 0.8 seconds, the axial thickness of 2.5 mm, a voltage of 120kV, a current of 350mAs ) to axial film the thoracic inlet to the base of the lungs under free breathing. The patient's body surface was longitudinally fitted with a 5 cm lead wire from the sternum to monitor the patient's respiratory status expressed with sinusoidal waveform, whereby every 10% was a 1‐time phase (0~90%). Next, maximum and minimum intensity projected images (MIP, MIN) and average intensity projected images (AIP) were obtained after the reconstruction of 10‐time phases using the respiration time‐phase fusion control technology.^,6^ Lastly, all the acquired images were uploaded to the Monaco TPS, and radiotherapy physicians (with more than five years of work experience) contoured tumor targets following the National Comprehensive Cancer Network‐China guidelines 2019 and subsequently reviewed by the radiotherapy chief physicians. They had a work experience of more than 10 years.

### 2.B Plan design and requirements

First, cases were prescribed with a VMAT of 6MeV photon energy, a fraction dose of 2 Gy, a fraction of 25, and a total amount of 50 Gy. Next, regarding the International Radiation Oncology Collaborative Group (RTOG) 1304 report, the OARs and tumor targets were dose‐constrained. Subsequently, the beam was designed using two arcs counter‐clockwise (150~300°) at a: grid spacing of 3 mm, beam margin of 5 mm, max.# control points per arc of 120, and a min.segment width of 6 mm. Consequently, we calculated the dose deposition to medium and adopted it for planning design. In this study, we planned to use the Monaco 5.11 clinical planning system (Swedish Elekta company) with HP Z820 server, 128G memory, and NVIDIA TESLA C2075 GPU mount to design the treatment plan. Lastly, the procedures were carried out using Versa HD™ clinical linear accelerator (Sweden) that was equipped with a collimator of Elekta Agility™ (80 pairs of leaves).

### 2.C Experimental design of inspiratory motion

**Fig. 1:**
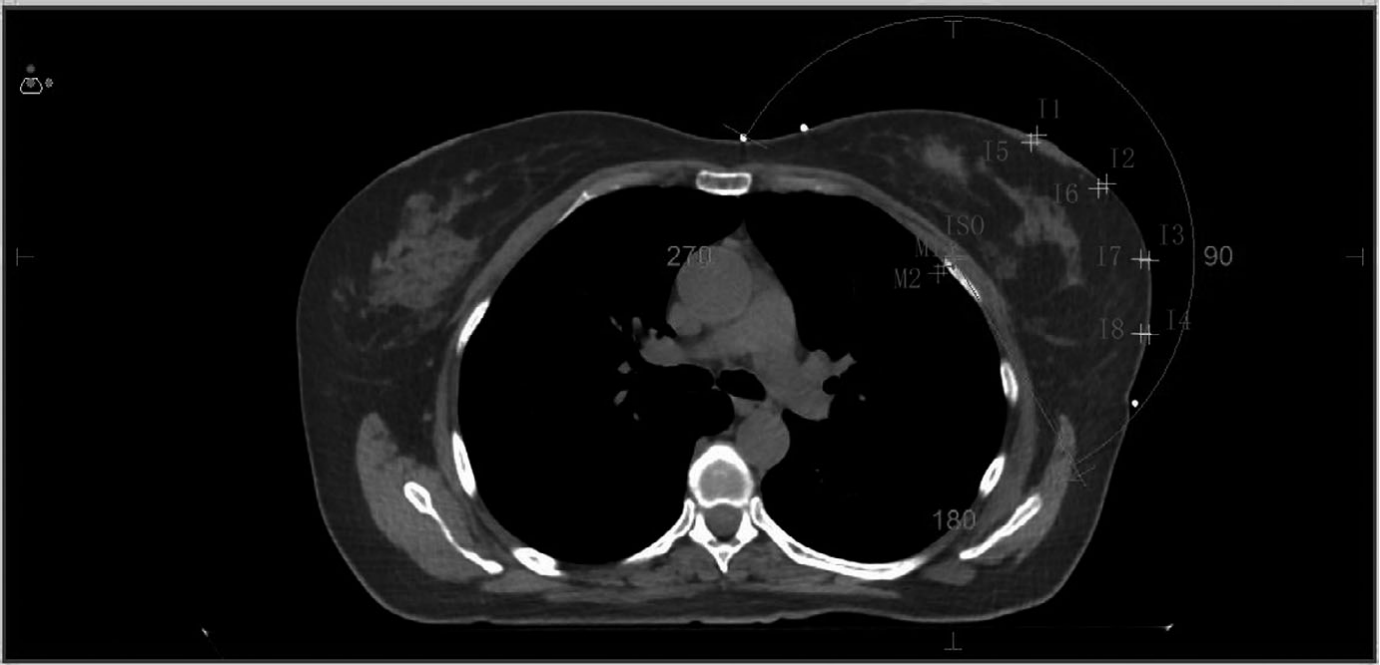
Dose measurement point and calculation center under simulated inspiratory condition. Note: I1~I4 are the dose measurement point on the skin surface, and I5~I8 are the dose measurement point of 3 mm under the skin. ISO is the general planning center, M1 and M2 are the calculating centers of simulate respiratory motions.

When selecting measurement points: we applied both interest points and markers to choose a range of 11~14 from skin boundary markers at different degrees such as 30°, 60°, 90°, and 120°. Also, a range of I5~I8 from 3 mm of the subcutaneous skin and a volume at a measurement point of 0.081cc,^8^, ^9^, ^10^ as presented in Figure 1, was used.

Comparison plan design: We kept all functions and parameters the same, used AFM 2.0 cm and not used AFM, respectively, the same group comparison plan was designed to evaluate the dose difference of tumor targets and OARs.

Evaluation of skin motility at the end of the inspiratory phase: The maximum and average motion distance of the inspiratory phase skin were assessed using the MIP, MIN, and AIP image fusion analysis of 4CDCT for all the 16 groups of patients with breast cancer.

Design of the simulated inspiratory motion plan: As illustrated in Figure 1, the average respiratory motion center M1 and the maximum motion distance center M2 were moved 2 and 5 mm in the X‐axis and Z‐axis directions with the isocentric facing away from the linac source. Took ISO as the planning center to complete the AFM and N‐AFM plans; took M1 as the planning center to complete the AF‐QA1 and NAF‐QA1 plans; took M2 as the planning center to complete the AF‐QA2 and NAF‐QA2 plans. The plans with M1 and M2 as the isocenter, used the original image as the QA calculation method of the verification phantom, so as to ensure that MLCs movement and #MU were exactly the same.

### 2.D Methods for validating and analyzing the plan

PTW solid water (30*30*10 cm) was scanned using a CT scan to establish a validation model. The AFM and N‐AFM plans from the same group were used to establish a model validation plan. In a similar batch as PTW solid water, the GAFCHROMIC^®^EBT3(Ashland Inc., Covington, KY, USA ID: 07221303) was placed parallel to the treatment surface table in solid water (5 cm above and below) and used for clinical plan validation. Lastly, the results of the film were scanned at the same time using an EPSON 10000XL scanner. Moreover, we validated that all the films were examined in the same direction according to the AAPM TG‐55 report,^10^ which used a dose Lab software for dose‐distribution and γ analysis of film scanning results.^12^, ^13^, ^14^

### 2.E Methods for statistical analysis of data

SPSS version 22.0 software was used for statistical data analysis of nonparametric variance using the Friedman test method. For all experiments, the probability value P < 0.05 was considered statistically significant.

## 3 RESULTS

### 3.A Analysis of inspiratory motion in breast cancer

**Fig. 2:**
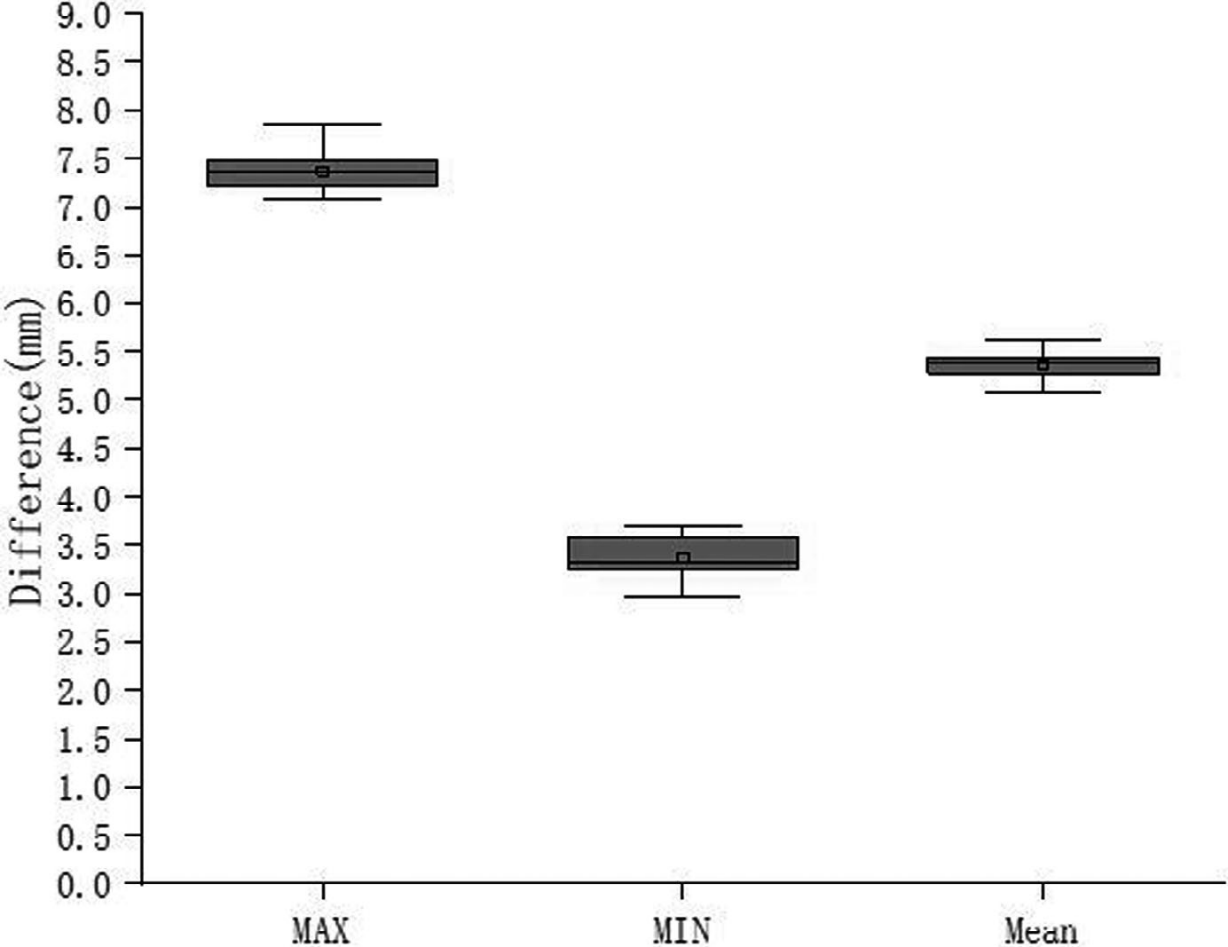
Analysis of maximum, minimum, and mean inspiratory motion in 16 breast cancers.

The MIP reconstruction and AVG images from the 4DCT scan of all the 16 breast cancer patients were imported into the Monaco TPS for image fusion. The maximum and minimum skin motion distances that belonged to each patient were recorded in the isocentric cross‐section. The top and minimum skin motion distances were 7.31 ± 0.45 and 3.42 ± 0.91 mm respectively. As illustrated in Figure 2, the average length was 5.53 ± 0.29 mm.^8^, ^9^, ^15^, ^16^

### 3.B Dose difference of tumor target and OARs between the AFM and N‐AFM groups

Table 1 shows the dose results of tumor target and OARs in plans from 16 groups of patients with the same parameters and functional conditions except the AFM setting changes. To read these results, we utilized the Monaco TPS DVH statistics. Here, we observed significant differences in the dose assessment results between PTV, Lung_Ipsi, and the skin form of both PTV and OARs dosimetry statistics.

**Table 1:** Influence of AFM and N‐AFM on dose results ($\bar{x}\pm s$).

	AFM	N ‐ AFM	*P*
PTV_V50Gy_ (%)	96.75 ± 0.172	94.64 ± 0.183	<0.05
PTV_D2%_ (Gy)	53.108 ± 1.001	53.981 ± 1.031	<0.05
PTV_Dmean_ (Gy)	51.823 ± 1.232	52.737 ± 1.314	<0.05
Lung_Ipsi_V20Gy_ (%)	15.01 ± 0.552	16.28 ± 0.563	<0.05
Lung_Ipsi_V5Gy_ (%)	49.678 ± 1.489	49.636 ± 1.532	0.587
Lung_Ipsi_Dmean_ (Gy)	9.487 ± 8.423	10.552 ± 8.963	<0.05
Lung_Contra_V5Gy_ (%)	14.467 ± 0.362	14.325 ± 0.341	0.632
Heart_Dmean_ (Gy)	8.021 ± 0.342	8.064 ± 0.372	0.657
Breast_Contra_V10Gy_ (%)	10.705 ± 0.228	10.856 ± 0.242	0.237
Body_Dmax_ (Gy)	54.031 ± 0.711	55.641 ± 0.725	<0.05

### 3.C Difference in collimator alignment between the AFM and the N‐AFM group at the same angle

**Fig. 3:**
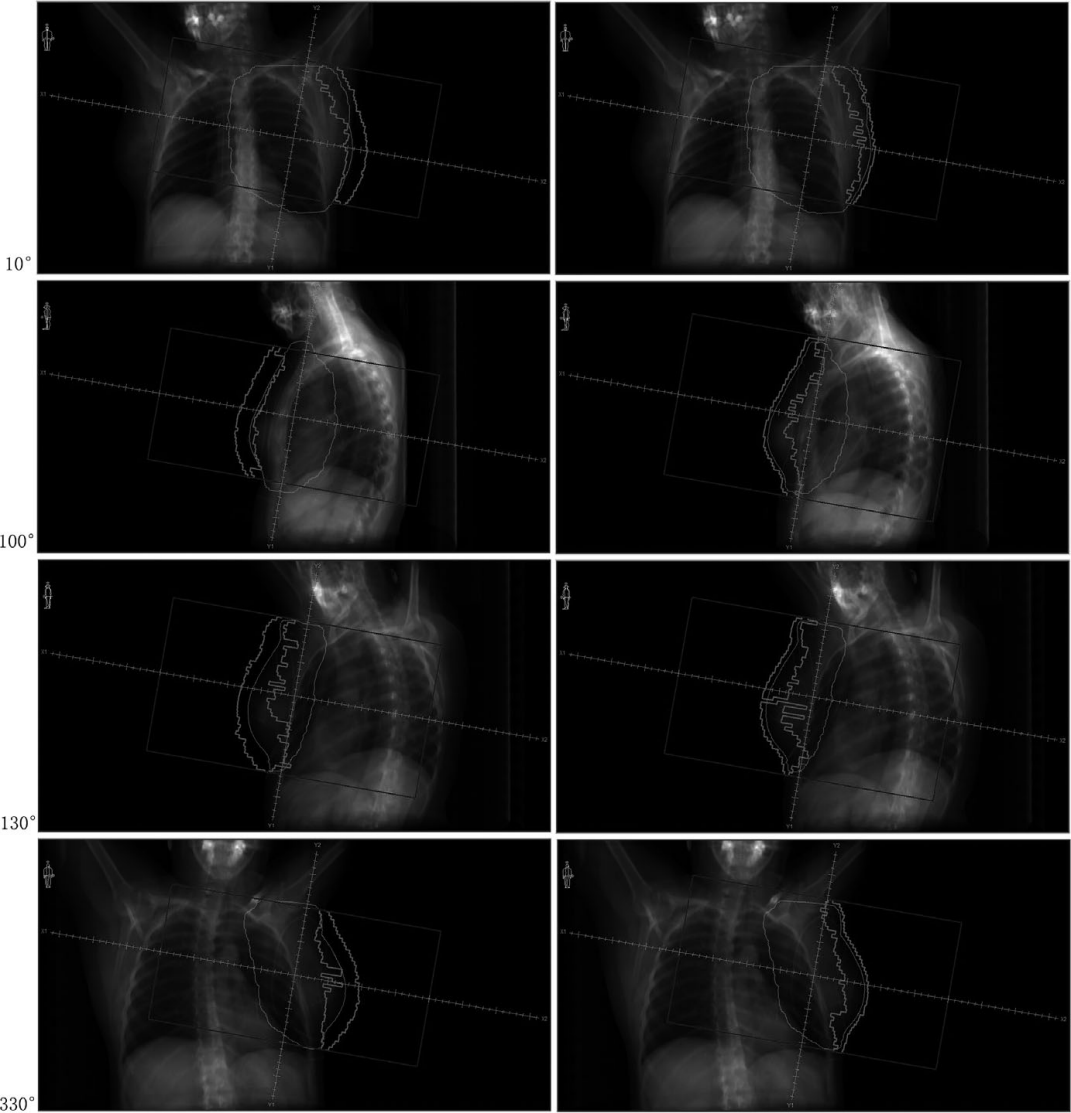
Difference in collimator alignment between AFM group and N‐AFM group. Note: The left side is the AFM experimental group, and the right side is N‐AFM experimental group.

Figure 3 shows the AFM and N‐AFM plans of the same group, whereby the following observation angles such as 10°, 100°, 130°, and 330°were selected, and subfields intercepted at the interface of BEV. Notably, at the four observation angles taken, the AFM group's collimator alignment formed an evident cavity volume outside the skin. In contrast, the N‐AFM group completed the leaves alignment according to the beam margin setting.

### 3.D Comparison of the X‐ray Voxel Monte Carlo (XVMC) characteristic parameters between the AFM and N‐AFM groups

**Fig. 4:**
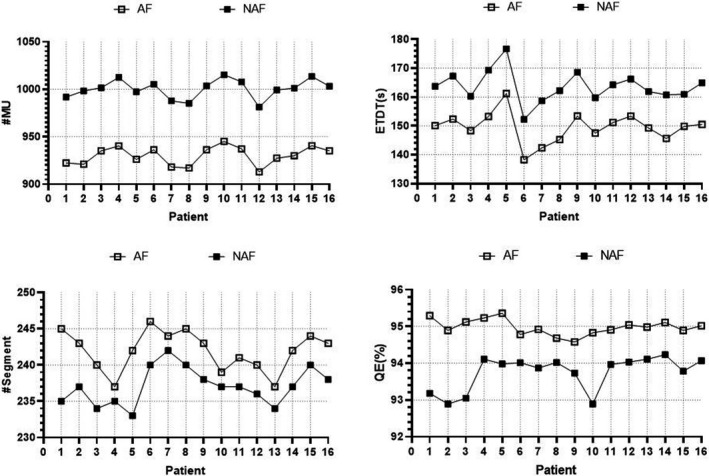
Comparison of XVMC characteristic parameters between AFM group and N‐AFM group. Note: #MU, ETDT, #segment, and QE from top left to bottom right.

As illustrated in Figure 4, calculation time (ETDT), #segments, #MU, and photon utilization (QE) were read using the Monaco TPS optimization console window and compared with each other. Here, it was observed that the comparison of #MU and ETDT showed that the N‐AFM group was substantially higher than the AFM group. In contrast with the QE, the AFM group was observed to be superior to the N‐AFM group. In comparison with the #segment, the AFM group was slightly higher than the N‐AFM group. However, both approached 240.

### 3.E Superficial dose difference between the AFM and N‐AFM groups under simulated inspiratory motion

**Fig. 5:**
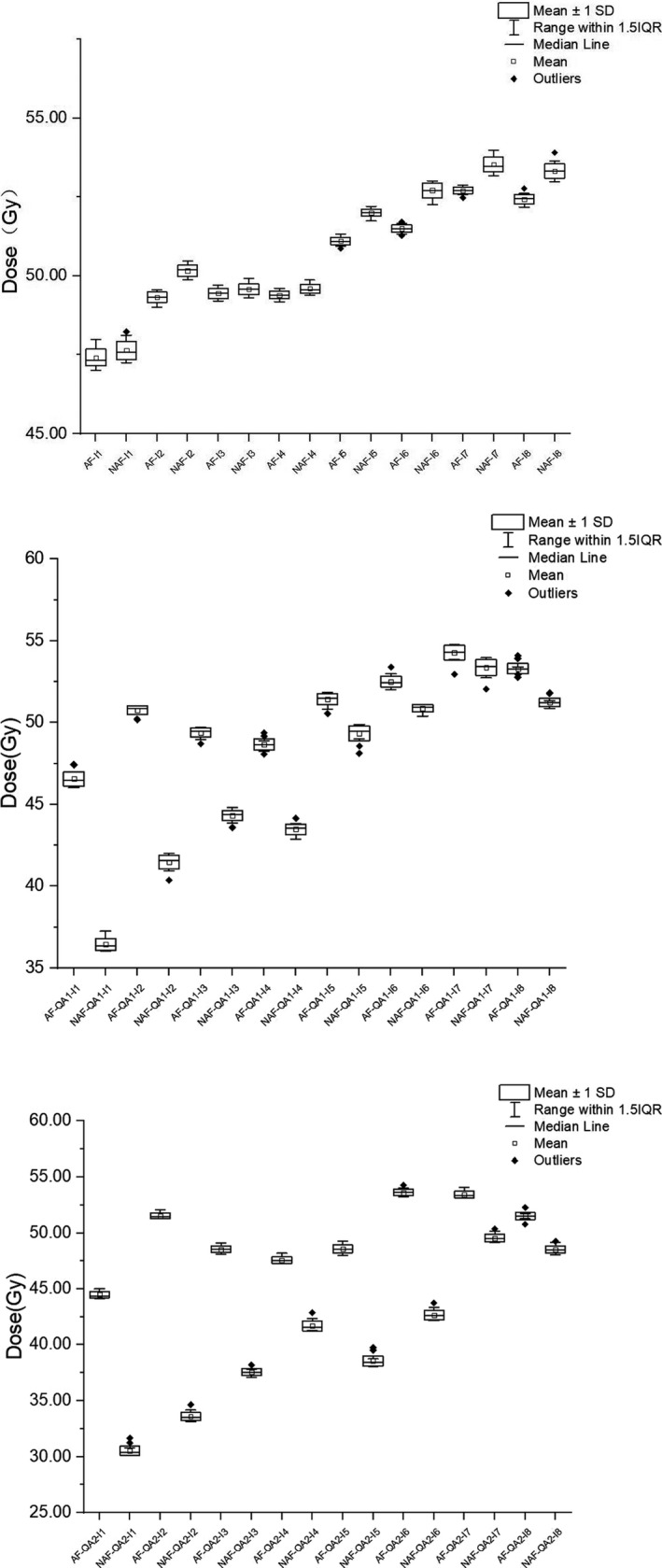
Dose differences of measurement points under the same respiration simulation conditions. Note: From top to bottom is AF and NAF, AF‐QA1 and NAF‐QA1, AF‐QA2, and NAF‐QA2.

**Fig. 6:**
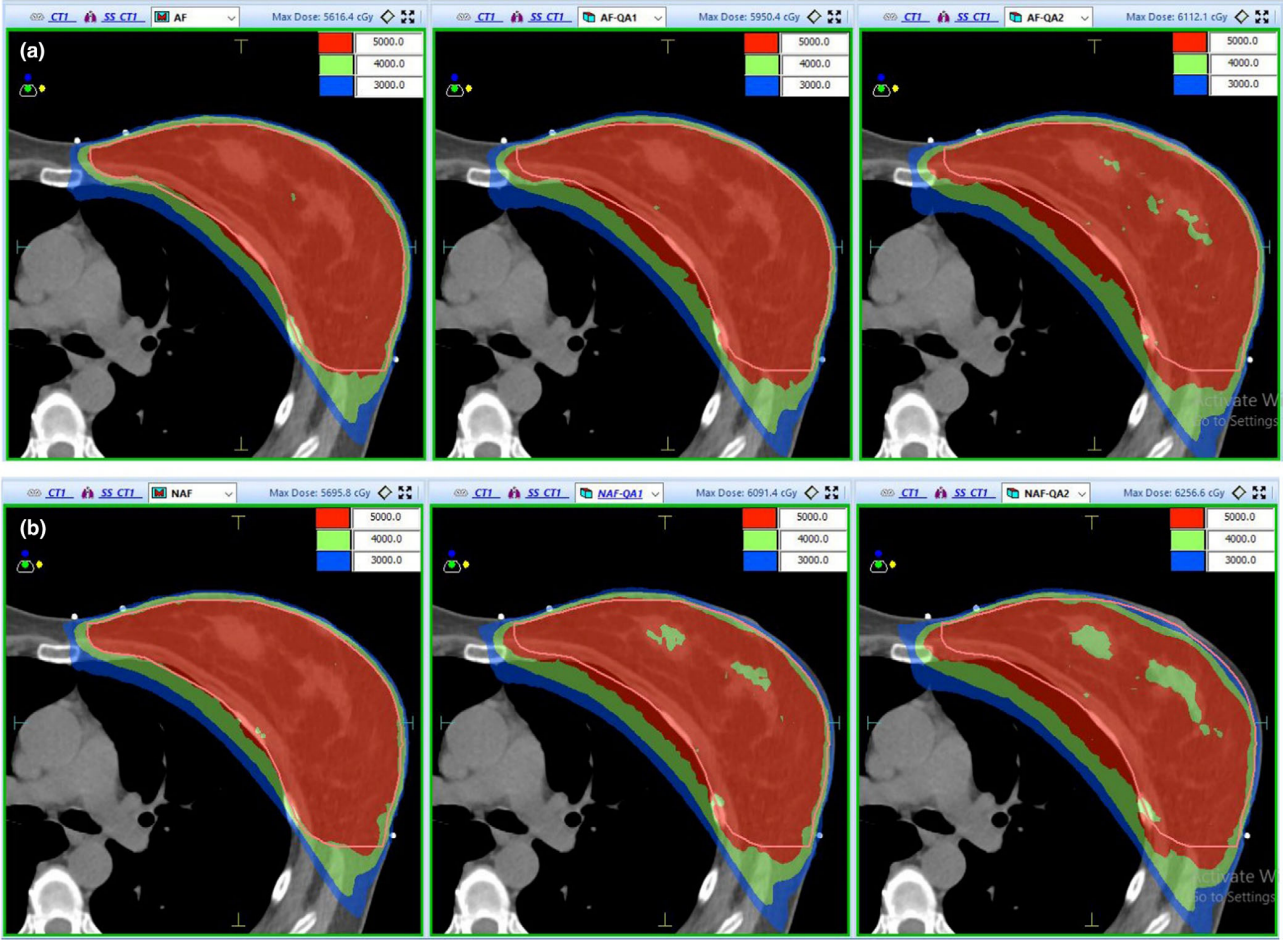
Dose nephogram distribution of AFM group and N‐AFM group. Note: Group a is the AFM experimental group, and group b is the N‐AFM experimental group.

The 16 breast cancer cases were all based on the superficial dose difference between the AFM and N‐AFM plans and images. The maximum and minimum motion distances of the skin were summarized according to the 4DCT scan. Here, the M1 and M2 results were taken as the dose calculation center (2 mm was moved in the direction of X and Z) with the isocentric facing away from the linac source; the M1 point moved 2.83 mm relative to the linac source according to the trigonometric formula. On the other hand, the M2 end moved 7.07 mm close to the linac source. The validating plans of AF‐QA1, AF‐QA2, NAF‐QA1, and NAF‐QA2 were established, and the dose calculation performed under the condition that the #MU and collimator alignment are similar. As illustrated in Figure 5, the dose point difference between the skin surface dose points I1~I4 and the 3 mm sub skin dose points I5~I8 were assessed under AFM and N‐AFM conditions. Lastly, the skin surface dose difference under the dose nephogram image was observed, as demonstrated in Figure 6.

In comparing measurement points of routine clinical plan in the AF and NAF group, the dose of each measurement point of the AF group was slightly lower than that of the NAF group. After simulating different respiratory motion distances in the AF‐QA1 and NAF‐QA1 & AF‐QA2 and NAF‐QA2 groups, it was observed that the dose of each measurement point in the AF group was significantly higher than that in the NAF group.

In the absence of inspiratory simulation, it was observed that after comparing the dose nephograms of the two groups under 50 Gy, 40 Gy, and 30 Gy, the dose difference between AFM and N‐AFM groups was small. However, as the inspiratory simulation distance increased, a significant dose drop was observed in the superficial breast area and the N‐AFM group's tumor target.

### 3.F Comparison of validation results between groups

**Fig. 7:**
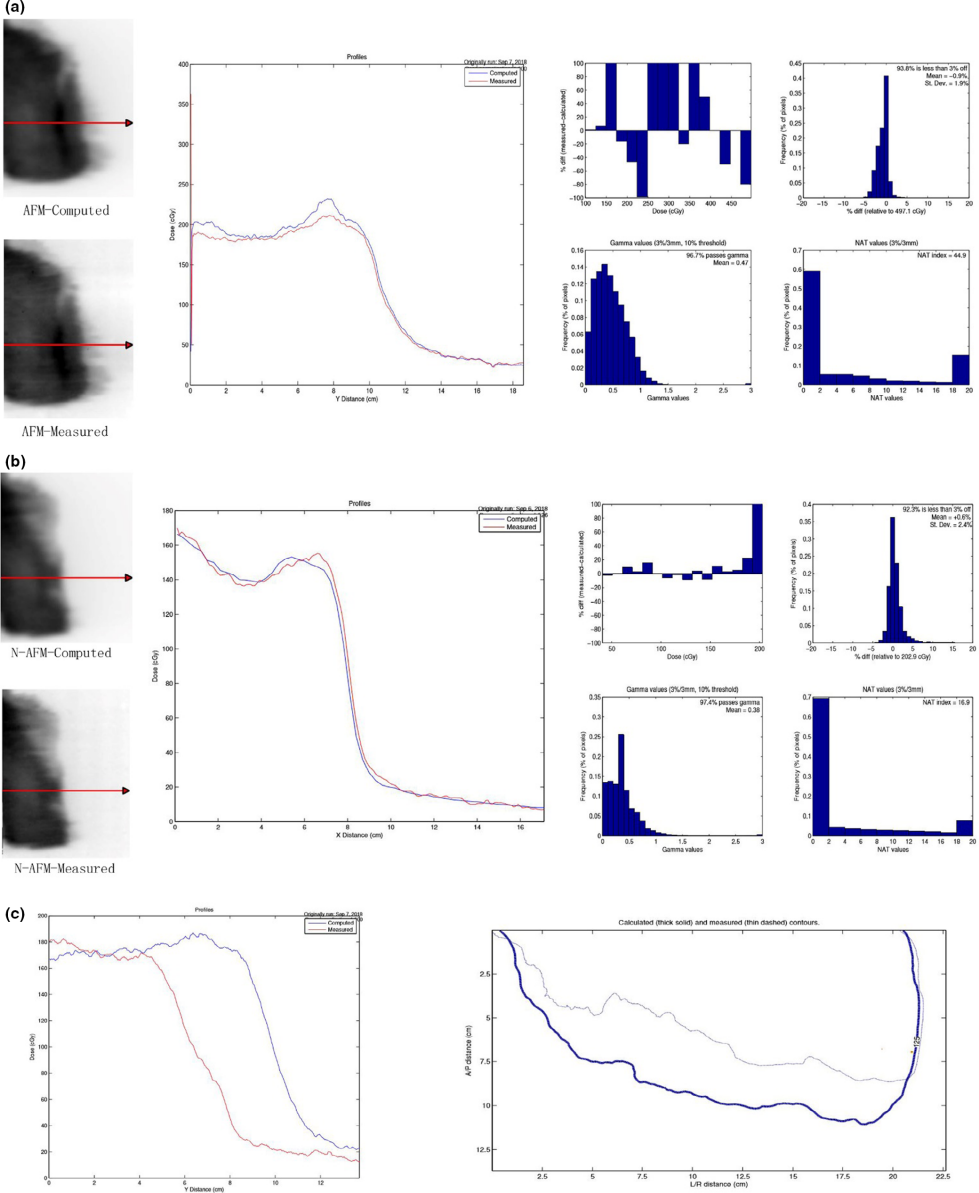
Comparison of film validation results between AFM and N‐AFM groups. Note: Group a is the film validation result of the AFM group, "Computed" is the output of the plan, and "Measured" is the film validation result. Group b is the result of film validation in the n‐AFM group, "Computed" is the output of the plan, and Measured results from film validation. Group c compares film measurement results between AFM and N‐AFM groups, "Computed" is the validation results of AFM, and "Measured" is the validation results of N‐AFM.

To validate the AFM and the N‐AFM plans in the same group, and EBT3 film was placed parallel to the treatment table surface in solid water (5 cm above and below). As illustrated in Figure 7, this film was then scanned using an EPSON 10000XL scanner, and the results were analyzed using doselab software.

In film validation (3, 3%), both the AFM and the N‐AFM groups had a pass rate of over 95%. However, the N‐AFM group's pass rate was slightly higher than that of the AFM group, hence, meeting the clinical application planning requirements. In the comparative analysis of film validation results between the AFM group and the N‐AFM group, the two groups' dose distribution at the far source end was the same. Still, the AFM group's dose distribution at the near‐source end was significantly higher than that of the N‐AFM group.

## 4 DISCUSSION

Although plans of the AFM and the N‐AFM groups were made under the same SSD, SOD, and XVMC functions and parameter optimization conditions, when comparing dose results of plans, it was observed that the AFM group was significantly superior to the N‐AFM group in the coverage volume of the prescribed dose in both the tumor target and the dose constraint of Lung_Ipsi. Generally, breast cancer's superficial area is located at the interface between air and skin tissue, and its main mechanisms during dose deposition are electron pollution and electron scattering accumulation. However, the difference in dose deposition was closely correlated to the beam field size, SSD, SOD, and tissue density.^17^, ^18^, ^19^, ^20^, ^21^ Then the collimator alignment of the beam from 2 plans at multiple but same angles was compared; it could be considered that the difference of beam field size was very small and negligible. In the AFM group, a 2 cm air cavity was established between the skin and the jaw. However, in the N‐AFM group, the MLC encoder between the skin and jaw was dominated by the intensity‐modulated plan. MLC leaves were aligned with the tumor target; hence, only a small or no air cavity was formed. This presence or absence of the air cavity was observed since there was no dose deposition of electrons in the air, and the air cavity was still filled with electrons because of field exposure. When the electrons from low‐density air collision reached the surface of the relatively high‐density human tissue and got scattered, the compton and electron pair effects occurred again between the electrons in the AFM group and the electrons in the cavity, and some of the electron energy would be deposited again in the superficial breast region. Therefore, a higher dose deposition was obtained in the superficial breast region in the AFM group than in the N‐AFM group. Under similar XVMC optimization function and parameter calculation conditions, it was observed that the AFM group obtained higher dose deposition in the superficial breast region. Thus, the dose optimization pressure in the superficial breast region was lower and less in‐target dose scattering compensation was necessary. In the N‐AFM group, the dose deposition in the superficial breast region was relatively low. Hence, to meet the target volume of prescription dose coverage in the target region, a relatively higher dose was needed to be formed inside the target region to carry out dose scattering compensation in the superficial breast region. Therefore, the maximum dose in the body, the maximum and average dose in the tumor target, the V_20,_ and the average dose of the lung tissue on the N‐AFM group plan's affected side were higher than those in the AFM group.

When comparing the XVMC characteristic parameters, it was easier to achieve the planning target of PTV prescription dose coverage volume in the AFM group because the electron scattering compensates the superficial breast region in the air cavity. Consequently, the dose optimization calculation in the AFM group was relatively simple in terms of the dose calculation complexity in the superficial breast region. Therefore, when comparing XVMC characteristic parameters between the two experimental groups, the AFM group applied less #MU and optimization time, and the QE% was higher. Besides, AFM increased the volume of the outer air cavity. Hence, the actual volume involved in the calculation was larger than that of the N‐AFM group, which was reflected in comparing #segments' parameters. With the increase in volume, the number of segments involved in the calculation increased slightly, but all of them tended to be close to the preset target of 240 control points.

I1~I4 on the skin surface and I5~I8 in the subcutaneous 3 mm were compared and evaluated in the AFM and N‐AFM static plan groups. It was observed that the point dose of I1‐I8 eight dose measurement points of AFM plan was lower than that of N‐AFM. This result was mainly due to the dose scattering compensation of AFM groups. The superficial breast group was higher than that of the N‐AFM group through the extracellular air cavity, and the required in‐target dose scattering compensation was less. As a result, the point dose comparison of the 8 points of the AFM static plan group was slightly lower than that of the N‐AFM group. Also, this result confirms the conclusion of comparing the dose results of the two groups above. The eight dose measurement points in the AFM‐QA1 and N‐AFM‐QA1 of minimum simulated inspiratory motion group, AFM‐QA2, and N‐AFM‐QA2 of maximum simulated inspiratory motion group, I1‐I4 on the surface, and I5‐I8 in subcutaneous 3 mm were compared and then evaluated. Here, each point dose of the AFM group was higher than the N‐AFM group. Dose difference of I1 ~ I4 4 points on the skin's surface compared with I5 ~ I8 4 points in subcutaneous 3 mm is more significant. Besides, the point dose difference of the eight measurement points (I1~I8) in the maximum simulated inspiratory motion group was more significant than that in the minimum. When the AFM was applied, we established measurement points of AFM QA1 and AFM QA2 plan in the 2 cm air cavity area in any simulated inspiratory motions.

The influence on the point dose mainly depends on the dosimetric change caused by SSD change due to the dose recalculation based on the new calculation center. The point doses of all measured points were close to or meet the dose standard of adequate breast cancer exposure. In the absence of AFM, under the condition of minimum simulate breathing motions, the N‐AFM‐QA1 plan was affected by dosimetric change caused by the shift in SSD and the shielded dose. This is because some parts of the leaves were too close to the tumor target. Therefore, in the four measurement points (I1~I4), there were obvious dose drops. The measured end could not meet the superficial area's sufficient irradiation requirements; due to the 5 mm beam margin's existence. The point doses of the four measurement points I5~I8 can still meet the standard of adequate breast cancer exposure. However, they also show the situation of dose drop. N‐AFM‐QA2 was a simulation of the maximum respiratory movement, thus a dose of some segments would be partial or total occlusions. Based on the point dose measurement results of I1~I4 measurement points, it is unable to meet the requirements of adequate exposure to breast cancer. We could see that the dose results of I5 and I6 could not meet the requirements of sufficient exposure, whereas I7 and I8 could still meet the requirements of sufficient exposure from dose measurement results of the four measurement points (I5 ~ I8). Therefore, there is a situation of dose‐angle response in the VMAT treatment plan for breast cancer. The dose in the lower breast region is higher than that in the upper breast region, and there is also a certain relationship with the dose control of the undiseased breast.

The point‐dose relationship between the static group plan and the simulated inspiratory group plan also obtained the same trend feedback as the measurement point‐dose comparison in the dose nephograms of 50 Gy, 40 Gy, and 30 Gy. In the AFM group, the static and minimum simulated inspiratory motion plan dose nephograms were almost the same. In the maximum simulated inspiratory motion plan, the dose data were close to the prescription dose in the skin and the subcutaneous superficial area. In the N‐AFM group, the static plan, minimum simulated inspiratory motion, and maximum simulated inspiratory motion plan in the superficial area of breast cancer showed a gradually obvious dose drop range. The dose of the maximum simulated inspiratory motion plan's superficial area was significantly unable to meet the requirements of adequate radiation exposure of breast cancer.

In evaluating plan validation results using EBT3 film, the pass rates of plan validation in the AFM group and the N‐AFM group all met the requirements of the clinical plan, and the N‐AFM group was slightly higher than the AFM group. The difference between plan projected results and film validation results was predominantly manifested in the rapidly descending dose area, such as the superficial area of breast cancer. In the profile comparison of the AFM group, it can be established that the measured results in the superficial breast region were higher than the plan projected results, so the actual electron scattering deposition dose in the air cavity area was higher than the estimated dose deposition in the superficial breast region using the XVMC algorithm. From the Profile comparison of the N‐AFM group, it can be found that the actual dose deposition in the superficial breast region is lower than the estimated dose deposition in the superficial breast region using the XVMC algorithm. In the group of AFM and N‐AFM validation film contrast analysis, the dose of difference between two groups was not significant in the initial distance of profile. Still, the AFM and N‐AFM film analysis results showed that the high dose drop‐off turning point appeared earlier in the N‐AFM group than in the AFM group. Thus, it was proved that there were many free electrons in the air cavity with AFM. Although the free electrons in the air cavity could not achieve the purpose of dose deposition, many free electrons would help improve the secondary scattering dose of electrons on the skin surface.

## 5 CONCLUSION

The AFM function of Elekta Monaco TPS has practical clinical application significance in breast‐conserving radiotherapy, which can effectively reduce the possibility of off‐target irradiation caused by inspiration‐induced motion, and achieve sufficient irradiation within a normal period of treatment, thus improving the efficacy and reducing the possibility of *in‐situ* recurrence of breast cancer.

## CONFLICT OF INTERESTS

The authors declare no conflict of interest.

## AUTHOR CONTRIBUTIONS

Lu Wang: setup experimental ideas and wrote papers. Gang Qiu: designed the experiment. Jianhe Yu and Qunhui Zhang: countered the cases and reviewed them. Li Man and Li Chen: analyzed and sorted out the experimental data. Xiaoxiao Zhang, Qun Ren and Hongxia Xu: carried out experiments and collated data. Xiaolong Hua: analyzed the experimental data and drafted the paper.

## Data Availability

The data that support the findings of this study are openly available in public repositories. All references are cited and the DOIs are attached. Please refer to section REFERENCES for details. The data that support the findings of this study are available from the corresponding author upon reasonable request. The data that support the findings of this study are available in the supplementary material of this article.
